# Immune Imprinting and Antigenic Drift as Determinants of SARS-CoV-2 Vaccine Performance

**DOI:** 10.7150/ijbs.130233

**Published:** 2026-06-11

**Authors:** Seung-Jae Lee, Jaehoon Bae, Sang Hoon Lee

**Affiliations:** 1 Functional Biomaterial Research Center, Korea Research Institute of Bioscience and Biotechnology, Jeongeup, 56212, Korea.; 2 Department of Applied Biotechnology, University of Science and Technology, Daejeon, 34113, Korea.; 3 Functional Food Research Institute, Industry-university Cooperation Foundation, Daegu Hanny University, Gyeongsan 38610, Korea.; 4 Division of Functional Food Research, Korea Food Research Institute, Wanju, 55365, Korea.; 5 Department of Food Biotechnology, University of Science and Technology, Daejeon, 34113, Korea.

He et al. presents a high-resolution longitudinal analysis of humoral immunity dynamics in southwest China, offering valuable insights into the evolving landscape of population-level immunity against SARS-CoV-2 1. By analyzing 3,128 monthly serum samples collected between December 2022 and April 2024, the study captures real-time changes in neutralizing antibody (NAb) responses as successive Omicron subvariants—including BA.5/BF.7, XBB.1.5, EG.5, and JN.1—sequentially replaced one another 1. This dataset provides a detailed temporal characterization and mapping of immune responses during the post-Omicron era.

A key finding of the study is that NAb responses are highly dynamic yet transient. Breakthrough infections during the BA.5/BF.7 wave elicited strong recall responses, reflecting activation of pre-existing immune memory 1. However, these elevated antibody levels declined rapidly within a few months, consistent with prior reports demonstrating waning humoral immunity following vaccination and infection 2. This observation reinforces the notion that infection-driven immunity alone is insufficient to sustain durable population-level protection.

Importantly, He et al. demonstrate that each successive variant wave induces a distinct immunological profile 1. While earlier Omicron variants such as BA.5 generated relatively broader neutralization responses, later subvariants—including JN.1—exhibited substantial immune escape, with significantly reduced neutralization sensitivity 1,3. These findings are consistent with recent studies showing that newly emerging Omicron lineages continue to accumulate mutations in spike protein antigenic sites, thereby enhancing antibody evasion 3,4.

One of the most significant implications of this study relates to immune imprinting. Prior exposure—either through vaccination or earlier infection—biases subsequent immune responses toward previously encountered epitopes 5. While this enhances early recall responses, it may simultaneously constrain the generation of broadly neutralizing antibodies against antigenically distinct variants 5. As a result, repeated exposure does not necessarily lead to improved cross-protective immunity, particularly in the context of rapidly evolving viral variants.

Notably, host-related factors such as age and sex appear to exert relatively limited influence compared with viral evolution itself. He et al. observed only modest differences across demographic groups, suggesting that antigenic drift has become the dominant determinant of immune escape at the population level 1. However, this interpretation should not imply that host-related factors are negligible. Demographic and clinical effects may become more pronounced in specific contexts, particularly among elderly individuals, immunocompromised patients, or individuals with comorbidities, and may also be influenced by prior infection and vaccination histories. This aligns with broader observations that viral evolution increasingly outpaces host immune adaptation.

Collectively, these findings highlight a critical mismatch between current vaccination strategies and the evolving antigenic landscape of SARS-CoV-2. Most vaccines remain based on ancestral or early Omicron spike sequences, which may no longer adequately represent circulating variants 6. Although updated bivalent formulations have shown improved immunogenicity, their ability to provide broad and durable protection remains limited 6.

The study by He et al. therefore raises an important question regarding the long-term sustainability of current vaccination strategies 1. One promising direction involves the development of next-generation vaccines targeting conserved viral epitopes. Such approaches aim to induce broadly neutralizing antibodies capable of recognizing diverse variants despite ongoing antigenic drift 7,8. Future vaccine development may therefore require platforms designed to broaden antigenic coverage beyond strain-specific spike updates. Promising approaches include mosaic coronavirus nanoparticles, pan-sarbecovirus vaccine formulations, mucosal or intranasal vaccines aimed at enhancing local respiratory immunity, and novel adjuvant strategies that may redirect memory B-cell responses toward newly introduced or conserved variant epitopes. In parallel, enhancing T cell-mediated immunity may provide more durable protection against severe disease, given its relative resistance to spike protein variation 9.

Beyond vaccine design, the work also underscores the importance of integrating real-time immunological data into public health frameworks. Current surveillance systems rely heavily on epidemiological indicators, which do not fully capture the dynamics of population immunity. Longitudinal neutralization profiling, as demonstrated by He et al., offers a more predictive framework for anticipating future variant waves and guiding adaptive vaccination strategies 1,10. Nevertheless, neutralizing antibody-based surveillance has inherent limitations. Although NAb titers provide a useful quantitative measure of antigenic mismatch and immune escape, they do not fully capture the complexity of protective immunity. Protection against severe COVID-19 is also strongly associated with cellular immune responses, including T cell-mediated immunity and memory B-cell responses, which may remain partially preserved even when neutralizing activity declines.

In conclusion, the findings of He et al. indicate that population-level immunity against SARS-CoV-2 is more transient and variant-dependent than previously assumed. The combined effects of rapid antibody waning, immune imprinting, and continuous antigenic drift challenge the long-term effectiveness of current vaccination strategies. Moving forward, more adaptive and forward-looking approaches that integrate immunological and evolutionary insights will be essential to mitigate the impact of future SARS-CoV-2 variants 10.

## Funding

This research was supported by the KRIBB Research Initiative Program (KGM1052612); a National Research Foundation of Korea (NRF) grant funded by the Ministry of Science and ICT (No. NRF-RS-2023-00208880); the Regional Innovation System & Education (RISE) program through the Jeonbuk RISE Center, funded by the Ministry of Education (MOE) and JeonBuk State, Republic of Korea (2025-RISE-13-WSU); and the Daegu Haany University Regional Innovation System & Education (RISE) Glocal Project Program [Global Joint Research on Traditional Medicine and K-Beauty] through the Gyeongsangbuk-do RISE Center, funded by the Ministry of Education (MOE) and Gyeongsangbuk-do, Republic of Korea (2026-RISE-15-110).

## Author contributions

S.J.L. and J.B. conceptualized the commentary, performed literature analysis, and drafted the manuscript. S.J.L., J.B., and S.H.L. contributed to discussion and interpretation. S.J.L. and S.H.L. supervised the work and critically reviewed and edited the manuscript. All authors have read and approved the final version of the manuscript.

## Figures and Tables

**Figure 1:**
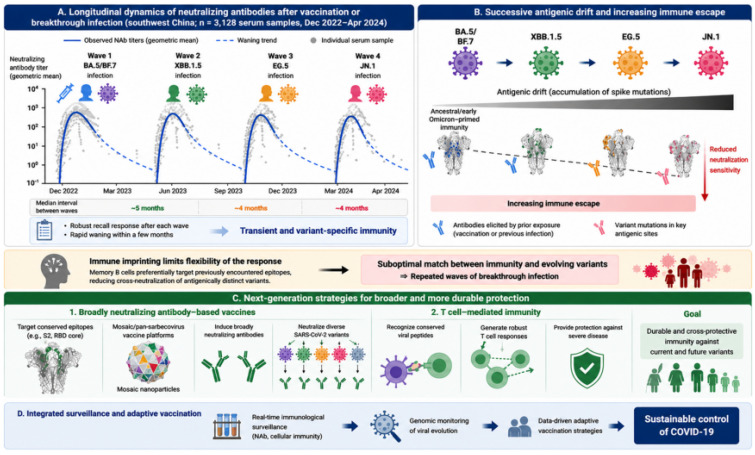
**Immune imprinting and antigenic drift shaping SARS-CoV-2 vaccine performance.** (A) Longitudinal dynamics of neutralizing antibody (NAb) responses following vaccination or breakthrough infection, based on findings from He et al. 1. Analysis of 3,128 serum samples collected between December 2022 and April 2024 shows repeated waves of antibody boosting followed by rapid waning, indicating transient and variant-specific immunity. (B) Sequential antigenic drift of SARS-CoV-2 Omicron subvariants (BA.5/BF.7, XBB.1.5, EG.5, JN.1) leading to progressive immune escape. Accumulation of spike protein mutations reduces neutralization sensitivity of pre-existing antibodies. (C) Immune imprinting effect, in which prior exposure biases antibody responses toward previously encountered epitopes, thereby limiting cross-neutralization against newly emerging variants. This results in a suboptimal match between host immunity and evolving viral variants. (D) Next-generation strategies to overcome immune escape, including vaccines targeting conserved epitopes to induce broadly neutralizing antibodies, enhancement of T cell-mediated immunity for durable protection, and integration of real-time immunological and genomic surveillance to guide adaptive vaccination strategies.
